# Integrating optical finger motion tracking with surface touch events

**DOI:** 10.3389/fpsyg.2015.00702

**Published:** 2015-06-02

**Authors:** Jennifer MacRitchie, Andrew P. McPherson

**Affiliations:** ^1^The MARCS Institute, University of Western SydneySydney, NSW, Australia; ^2^Conservatorio della Svizzera Italiana, Scuola Universitaria di Musica, The University of Applied Sciences and Arts of Southern SwitzerlandLugano, Switzerland; ^3^Centre for Digital Music, School of Electronic Engineering and Computer Science, Queen Mary University of LondonLondon, UK

**Keywords:** motion capture, capacitive sensing, touch, performance analysis, human-computer interaction, piano performance

## Abstract

This paper presents a method of integrating two contrasting sensor systems for studying human interaction with a mechanical system, using piano performance as the case study. Piano technique requires both precise small-scale motion of fingers on the key surfaces and planned large-scale movement of the hands and arms. Where studies of performance often focus on one of these scales in isolation, this paper investigates the relationship between them. Two sensor systems were installed on an acoustic grand piano: a monocular high-speed camera tracking the position of painted markers on the hands, and capacitive touch sensors attach to the key surfaces which measure the location of finger-key contacts. This paper highlights a method of fusing the data from these systems, including temporal and spatial alignment, segmentation into notes and automatic fingering annotation. Three case studies demonstrate the utility of the multi-sensor data: analysis of finger flexion or extension based on touch and camera marker location, timing analysis of finger-key contact preceding and following key presses, and characterization of individual finger movements in the transitions between successive key presses. Piano performance is the focus of this paper, but the sensor method could equally apply to other fine motor control scenarios, with applications to human-computer interaction.

## 1. Introduction

Many human-machine interactions require large-scale positioning of the hand and arm coupled with fine motor control of each finger. Most interfaces, whether they are discrete mechanical objects (computer keyboards, piano keys) or continuous surfaces (smartphones, trackpads), directly sense only the end result of an action: the arrival of the finger at a particular location. However, understanding the human actions that go into using these systems requires knowledge of a larger biomechanical context. For example, piano teachers devote substantial attention to the motion of hands, wrist, arms, and torso; few if any teachers would instruct a student based on finger movements alone. Design of human-computer interfaces is also influenced by factors beyond what can be directly sensed, for instance in touchscreen interfaces for very large smartphones, where the maximum extension of the hand needs to be considered in positioning controls onscreen.

Recent human-computer interaction research, especially on mobile devices, has explored actions taking place in the space around a device as well as the movement of the device itself (Lane et al., 2010). Augmented interaction in free space has been achieved through proximity sensors (Kratz and Rohs, 2009), ultrasonic rangefingers (Przybyla et al., 2014), and electromagnetic field sensing (Zimmerman et al., 1995; Cohn et al., 2012) among other means. In the musical domain, augmented keyboard instruments have been developed using depth cameras to measure hand movements in the air above the keys and control alterations to the sound (Hadjakos, 2012; Yang and Essl, 2014).

Whenever multiple sensors are used simultaneously, combining and synchronizing the data becomes an important consideration. Examples are many within multi-camera sensing, including combinations of RGB and depth camera data (Ohn-Bar and Trivedi, 2014), combinations of thermal and depth camera data (Saba et al., 2012), and homogeneous multi-camera systems as widely used in motion capture equipment. Touchscreens have likewise been combined with other sensor modalities, including inertial motion sensors (Xu et al., 2012), proximity sensors (Butler et al., 2008), depth cameras (single or multiple) (Wilson and Benko, 2010; Kim et al., 2014), and tangible object sensing (Marquardt et al., 2011; Nunes et al., 2014).

Tabletop interaction is a particular focus of sensor combination efforts; Marquardt et al. define a “continuous interaction space of new actions combining sensors integrated into a surface and motion capture above that surface” (Marquardt et al., 2011). Examples include grasping gestures which begin on the surface and continuing into the space above it, hovering above the surface, and “extended reach” gestures by pointing to areas beyond the reach of the hand. Sensor fusion techniques have also been used in digital musical instruments, combining multiple sensor modalities on the same object for better accuracy (Medeiros and Wanderley, 2014) or integrating sensors with audio for more robust feature extraction (Pardue et al., 2014).

A challenge occurs when discrete and continuous sensors are combined. Discrete sensors (e.g., individual piano or keyboard keys) are suited to recognizing individual actions, but the continuous data (e.g., motion capture of the hands) must be segmented to determine which parts are associated with which actions. Conversely, higher-level motor planning cannot easily be deduced from discrete sensors unless each action is first aligned with a continuous data stream. The proper alignment and segmentation may not be obvious, especially when the sensors do not measure the same object. In the case of combining touch and camera data, the surface touch is inherently occluded from the camera by the back of the finger and hand, and the motor actions associated with a particular key press may both precede and follow the finger-key contact.

This paper highlights two facets of multi-sensor performance measurement. The first concerns the general question of combining heterogeneous data sources, aligning them in time and space and segmenting continuous data into discrete events. The second examines the research questions about human performance which can be addressed with this method. Piano performance will be the case study, with a focus on finger-key interaction, however similar techniques could be applied to other domains.

### 1.1. Sensor technologies measuring finger-key interactions

Piano performance studies focus on measurement of either the performer's body (particularly hands and fingers) or the mechanical keys themselves. Sensor devices can be categorized by whether they are measuring continuous movement across multiple notes or discrete per-note events. A variety of sensors have been used to measure continuous movement, including datagloves (Furuya et al., 2011a; Furuya and Soechting, 2012), accelerometers, electrogoniometers (also used to study typing performance Treaster and Marras, 2000), motion capture with active or passive markers (Goebl and Palmer, 2008), and markerless image processing (see Metcalf et al., 2014; MacRitchie, 2015 for reviews of these technologies). MIDI (Musical Instrument Digital Interface) data is the most common method of measuring individual notes (see Minetti et al., 2007 for a representative setup on the acoustic piano), but studies also focus on continuous key angle (Bernays and Traube, 2012; McPherson and Kim, 2012), force measurements (Parlitz et al., 1998; Kinoshita et al., 2007), and touch location on the key surface (McPherson et al., 2013).

Although tools exist to analyse body movements solely from video images in music and dance performances (Jensenius, 2007), many of the sensor systems relating to hand and finger movement are limited by high cost and the need for specialist knowledge to use them (MacRitchie, 2015). This means that performance studies are often conducted in a laboratory environment, and in particular, studies of pianists' movements are conducted on an electronic keyboard so as to acquire precise data on the onset and release of keypresses. The technologies described in this paper are designed to function on any piano, acoustic or electronic, encouraging use outside the laboratory.

The primary contribution of this paper is the combination of specific complementary technologies to measure finger-key interaction. Previous instances of sensor combination include Dalla Bella and Palmer's study where finger height measurements were compared to MIDI note onset times (Dalla Bella and Palmer, 2011), and the multimodal system by Grosshauser and colleagues incorporating accelerometers, torque and tilt sensors on the hand (Grosshauser et al., 2012). Continuous data from each sensor is visualized alongside MIDI onsets and releases which demonstrate changes in finger movement as the key is pressed and released. Kinoshita and colleagues use LED motion capture combined with a force transducer on the key surface (Kinoshita et al., 2007). Analysing and integrating combinations of sensor data can be difficult, so many studies are limited to using average or maximum measurements of each source for each touch event, or visualizing the raw data. What is needed is a clearer relationship between the continuous motion of the body and the specific touch events it produces. This paper demonstrates not only a combination of sensors, but methods for combining the data to address new research questions in human performance.

### 1.2. Biomechanical studies of finger-key interaction across multiple keypresses

Studies of finger motion near the key surface typically focus on the vertical movement of the finger in relation to timings of key press events; see (Furuya and Altenmüller, 2013) for a review of the biomechanical literature in this area and (MacRitchie, 2015) for a general review of the study of piano touch. The velocity hammer-string collision, and therefore the key press velocity, is the main factor in determining the tone quality of an individual note (Furuya et al., 2010; Goebl et al., 2014), though recent studies have found that impact noises between finger and key, or between key and key-bed, are also perceptible (Goebl et al., 2014). This suggests that apparently redundant finger movements may have a purpose in shaping perception.

Other piano studies focus on arm movement, showing that it tends to be circular rather than vertical, and that is highly influenced by the layout of the keys being pressed (Engel et al., 1997). Studies of finger force on the keys show different temporal profiles for different types of touch (Furuya and Kinoshita, 2008) and that for the same passages, novices exert more and longer force on the keys than experts (Parlitz et al., 1998). The force sensors in those studies consider only the vertical axis of motion, though Grosshauser and Tröster (2013) demonstrate a key-top matrix force sensor showing the location of the applied force. By contrast, the capacitive key-top sensors used in this paper (McPherson, 2012) measure surface touch location rather than force, and our measurements and analyses here focus primarily on the horizontal plane of motion (i.e., along the key surfaces) rather than finger height above the keys or pressure into the key-bed.

Recent typing studies focus on the changing wrist angles used while performing consecutive keypresses. Individual differences were found concerning tendon travel that were only partially explained by gender and hand anthropometry (Treaster and Marras, 2000). This may be indicative of an individualized pattern of keypresses in typing that can also be characterized by keystroke latencies (Joyce and Gupta, 1990). This individualized fingerprint has also been found in piano performance in the timing of scale passages (van Vugt et al., 2013). Measurements from these devices are often taken in terms of maximum key force, or maximum angle velocity at a particular keypress in order to relate the continuous movement with the keypress, however, in reality, although the keypress movement is strictly vertical, the human interaction with it in the context of piano performance is three-dimensional.

The pedagogical piano literature suggests many different approaches to the rich, complex interactions between the hand motion and the key press event which remain understudied (MacRitchie, 2015). For example, Berman (2000) suggests that in order to achieve a “warm, singing” sound, a pianist must use flexed fingers, whereas curved fingers may be used for good articulation. Newer methods taking advice from anatomical and biomechanical studies suggest that pianists should make natural “curved” movements, and use gravity in order to effect a more efficient downswing of the arm (James, 2012).

What is required to elucidate the finger-key interaction is a combination of sensors working in tandem to reveal the anticipatory motions of intent, the touch event throughout the length of finger-key contact, and the movement away from the key surface toward the next touch event.

## 2. Method

The proposed method focuses on the integration of two novel sensor devices, the alignment of the data recorded by each, and the valuable questions that can be answered from a proposed set of analyses.

### 2.1. Devices

**Figure 1:**
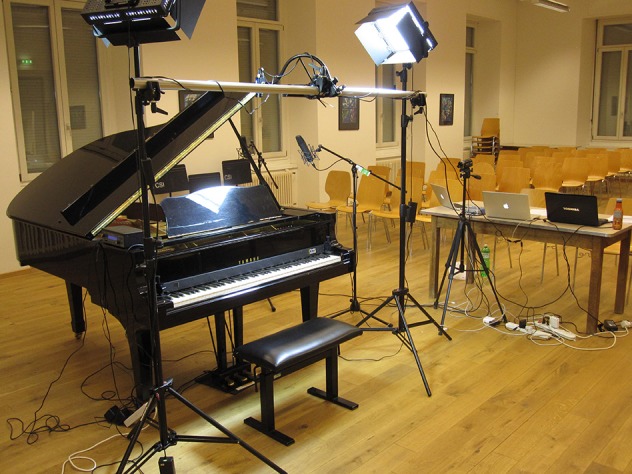
**Setup of three sensors on a Yamaha C5 grand piano: monocular high-speed camera (scaffolding at top, facing downward), TouchKeys sensors (on key surfaces); Moog PianoBar (at back of keyboard)**.

The setup includes three particular elements: a monocular motion capture system using a single RGB camera suspended above the keyboard, capacitive key sensors attached to the key surface of the piano, and an infrared MIDI sensor which sits at the back of the keys. The motion capture system and the capacitive key sensors collect data on the location of the markers and touch events, respectively. Location is measured in two axes; in this paper, *X* refers to the bass-to-treble axis from left to right along the keyboard, with larger values in the treble direction; *Y* refers to the lengthwise axis of the keys, with lower values toward the player and higher values toward the fallboard of the piano. These devices are seen in a setup together in Figure 1. Due to processing requirements of the motion capture camera, data is acquired through a separate computer to that of the other devices. Data was collected on this particular occasion on a Yamaha C5 grand piano situated in a small concert hall in a music conservatory.

#### 2.1.1. Monocular motion capture

The monocular image-processing based system detailed in MacRitchie and Bailey (2013) involves tracking colored markers from a single RGB camera with an aerial viewpoint. Cameras with increased frame-rates above the standard 25 fps are preferred, operating on a reduced region of interest. Using a single camera and passive markers reduces the cost and processing power required to run the system, allowing it to be used in a variety of environments, unrestricted in terms of the instrument to be used, or the venue in which the participant is recorded. This 2D capture system estimates the depth of markers by monitoring the XY distance changes of particular reference markers at the palm, however, the strengths of this system are in the XY data capturing at the plane of the keyboard. In using markers to track the movement of each particular finger (two markers on the wrist, and markers on each of the metacarpophalangeal, proximal interphalangeal, and distal interphalangeal joints), the motion capture system can acquire accurate data on the flexion/extension of each finger joint in terms of the associated markers' changing distances. Modifications from the original hardware of the system in MacRitchie and Bailey (2013) include use of bright stage-paint for markers eliminating the need for the UV darklight, and use of a different camera to the original, allowing capture rates of 117 fps instead of the original 60 fps. Modifications to the software of the system consist of algorithms to apply camera undistortion and piano key detection.

The original system processed images directly from the camera stream or video file, however these were subject to distortion due to the nature of the camera lens. Using calibration techniques available in the Intel OpenCV library (version 2.4.6), the camera's distortion matrix is calculated and applied in order to undistort both the captured image and the marker positions. Currently this stage is performed post-processing.

In order to align the positional data with the identified key in terms of a keypress, a further additional stage has been added post-processing and after the undistortion process. Detection is performed on the undistorted image of keyboard, with the hands not present. Hough transforms are used to identify the three horizontal boundaries of the keyboard area: the top of the keyboard, the bottom of the black keys, and the bottom of the white keys. Color thresholding on the grayscale background image and blob detection identifies black blobs within this area (the black keys). Once the user identifies the position of key C4 by clicking on the key area, coordinates of the white keys in between these black keys are calculated and a set of piano key polygons are saved identifying each key.

#### 2.1.2. Capacitive key-sensing devices

**Figure 2:**
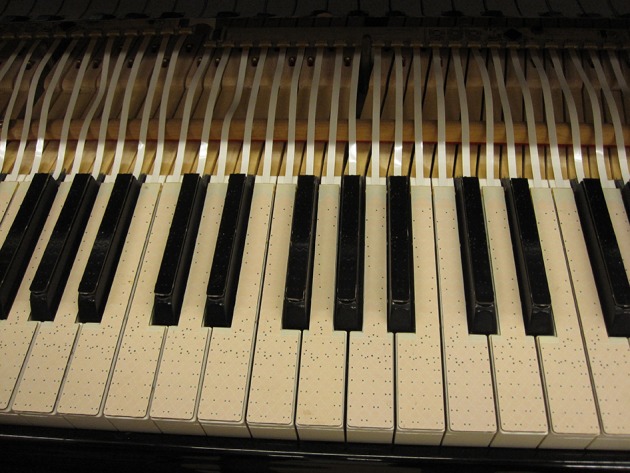
**TouchKeys capacitive touch sensors attached to key surfaces on a Yamaha C5 grand piano**. Setup shown here with piano fallboard removed; cables route underneath fallboard in performance.

Capacitive touch sensors were affixed to the surface of each piano key (Figure 2). These *TouchKeys* sensors (McPherson, 2012) measure the location and contact area of fingers on the key surfaces. The TouchKeys measure XY position on the entire surface of the black keys and on the front 6 cm of the white keys (encompassing the entire wide front part of the key, plus 1 cm of the narrow part behind it). The rear part of each white key measures Y position only. Because the finger is constrained in this region by the neighboring black keys, this is not a significant limitation.

Spatial resolution of the sensors is less than 0.1 mm in each axis, and the samples are taken every 5 ms for each key. The Y location can be measured for up to three touches per key (for example, multiple fingers on the key during a finger substitution), with one average X location for all touches. Contact area is most sensitive to the difference between touches with the fingertip vs. the pad of the finger. No pressure is needed to activate the sensor, and touch data is measured whether or not a key is pressed down.

The sensors are affixed to the keys using strong but removable double-sided adhesive. Each sensor is connected via a flat flexible cable; the cables are routed under the fallboard of the piano to controller boards resting inside the instrument. The controllers are attached to a computer via USB. Data frames from the TouchKeys are marked with MIDI note numbers, a timestamp in milliseconds generated by the internal device clock, position (8 bits for X, up to 12 bits for Y), and contact area (8 bits). For the data recorded with the particular setup in Figure 1, raw touch data was logged to a file for later analysis.

The white key sensors weigh 5g (including adhesive); the black key sensors weigh 2g. The sensors add 1.6 mm to the height of each key surface, but because the height is the same for each key, the relative heights of black vs. white keys are unchanged. When using these sensors in the setup described, an informal observation made by both the authors and the pianists was that the addition of the sensors was not found to noticeably change the action of the piano. Relative to the standard key tops, the TouchKeys sensors have squarer, less rounded edges. Pianists reported noticing this difference, particularly on the sides of the black keys, but stated that it did not significantly inhibit their performances after a certain practice period.

#### 2.1.3. Infrared MIDI sensors

In order to measure key motion, there are two types of sensor available. The Moog PianoBar (Figure 1) is a commercial device (now discontinued) which generates MIDI information from the keys using optical reflectance sensing on the white keys and beam interruption sensing on the black keys (McPherson, 2013). Experimentally, we found that the timing of MIDI messages from the PianoBar was sufficiently accurate, but that the velocity measurements of key presses were unreliable. Velocity is therefore not used in our analyses. Magnetic pickups record the motion of the left (*una corda*) and right (damper) pedals, producing a binary on/off value for each pedal. The PianoBar occupies 1.3 cm at the back of the keyboard.

**Figure 3:**
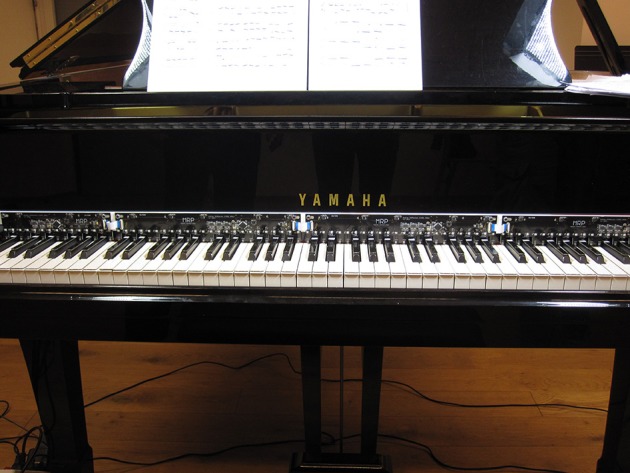
**Continuous key angle infrared sensor on a Yamaha C5 grand piano**.

An alternative to the PianoBar is an experimental continuous key-angle scanner (McPherson, 2013) which uses reflectance sensing on both black and white keys (Figure 3). Continuous key angle is sampled at 1 kHz per key, with the effective resolution depending on the amount of reflected light (typically between 7 and 10 bits, depending on distance). This sensor occupies 7 mm at the back of the keyboard.

The bulk of the measurements collected with this setup are made with the PianoBar because of its proven track record and because MIDI is sufficient for most analyses. However, the continuous scanner offers opportunities for more detailed future analyses of keyboard touch.

### 2.2. Data alignment

To measure any one performance, our method uses the systems in Section 2.1 together, such that each performance is recorded using camera tracking, touch sensing and key motion measurement. Each device operates with a different set of temporal and spatial coordinates, so alignment of the data sources is the first step in any analysis.

#### 2.2.1. Timestamps and sampling rates

Time alignment of camera, touch, and MIDI data sources is challenging because each device has its own clock and its own frame rate. The camera operates at 117 Hz, the TouchKeys at 200 Hz. MIDI data has no fixed frame rate, but the internal operation of the Moog PianoBar suggests that the sampling rate of the optical sensors is approximately 600 Hz (McPherson, 2013). The continuous key-angle scanner, used as an alternative to the PianoBar, has a frame rate of 1000 Hz, but it is clocked independently from the TouchKeys, allowing clock drift.

Computer system timestamps alone are insufficient for time alignment. First, the high-speed camera is operated on a different computer from the touch and MIDI (CPU and drive speed limitations prevent all three from operating together). Second, the system timestamp reflects when the data is received by the logging program, not when it is generated. For USB devices such as the TouchKeys, the operating system USB drivers can introduce significant and unpredictable latency.

First, new timestamps are generated for each camera frame based on the known sample rate. Next, TouchKeys timestamps are regenerated based on the frame numbers in milliseconds recorded by the hardware. Because the clock in the TouchKeys microcontroller may drift from the computer system clock, 1 ms as measured by the TouchKeys may differ from 1 ms as measured by the computer. The first and last frame numbers and the first and last computer system timestamps are analyzed: the difference in computer time divided by the difference in frame numbers gives the actual frame rate. Typically, the frame rate reported by the TouchKeys was accurate to within 0.01% of the computer clock.

The new TouchKeys timestamps are calculated relative to the first timestamp recorded by the computer clock. Subtracting the original from the regenerated timestamps gives the relative latency introduced by USB; in the performances we analyzed, we found that this latency could reach 400 ms for some frames, though on average the difference was less than 1 ms. MIDI timestamps are left unchanged, as there is no source except the computer clock to record these.

Next, camera and MIDI timestamps are aligned. The onset time of the first three notes is identified visually from the camera data. These times are compared to the MIDI timestamps for those three notes, and time offsets are calculated for each one. The mean of the three offsets is then added to all MIDI and touch timestamps. The final result is a single set of timestamps aligned to the camera, with 0 s marking the first recorded camera frame.

#### 2.2.2. Spatial coordinates

Two spatial alignments need to be performed: camera pixel coordinates need to be associated with individual keys (MIDI note numbers), and touch sensor locations need to be aligned to camera coordinates.

The camera tracking software (Section 2.1.1) generates a file containing polygons in pixel coordinates for each piano key (4 vertices for the black keys, up to 8 vertices for the white keys). Based on these polygons, every frame of marker data is assigned a MIDI note based on which polygon it falls inside. If a marker on a finger is associated with a MIDI note, it does not necessarily mean that finger has played the note, only that that particular part of the hand is above the key; for example, the distal and proximal markers on a finger might be associated with different MIDI notes depending on the finger angle. When a marker falls in front of the keyboard (low *Y*-values), MIDI notes are assigned based on the X position, indicating which white key the marker is closest to.

Each touch sensor frame is marked with a MIDI note and positions relative to the length of the key (0–1 in each axis). These coordinates are converted to camera pixel coordinates using the stored polygons for each key. The result of these steps is touch data aligned in time and space with camera marker data, allowing comparative analysis of the point of finger-key contact with respect to the positions of each of the joints of the hand.

#### 2.2.3. Automatic fingering detection

**Figure 4:**
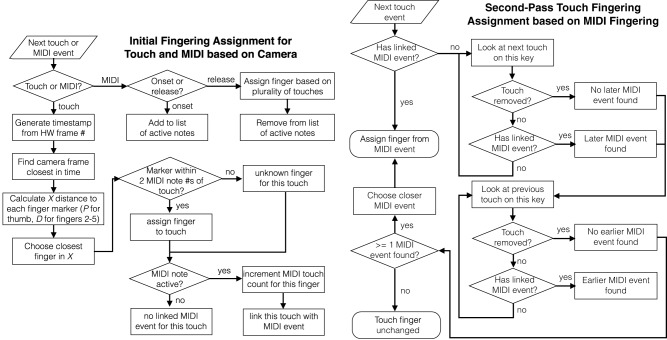
**Flowchart for automatic fingering assignment to touch and MIDI data**. **Left**: Initial fingering of touch and MIDI frames based on camera data, and association of touches with MIDI events. **Right**: Second-pass touch fingering correction based on assigned MIDI fingerings.

The touch sensors capture finger-key contacts, but cannot themselves distinguish which finger pressed the key. By using the camera data, touch frames are automatically assigned to fingers, as shown in Figure 4. Given a touch frame aligned in space and time, the temporally closest camera frame is found, and the distal markers for each finger are examined. The finger whose marker has the smallest *X* distance from the touch is identified as the finger which produced the touch.

Fingering is also automatically assigned to each MIDI note. While a MIDI note is active, the fingers associated with each touch on that note are counted; when the note is released, the finger which generates the plurality of touches is chosen as the finger for that MIDI note.

Once fingerings have been generated for all MIDI notes, a second pass through the touch data corrects any erroneous fingerings. Touches which take place while a key is held down are assigned the fingering for that MIDI note. However, touch frames also precede and follow most key press events. For touch frames which occur outside the duration of a key press, an uninterrupted sequence of touch frames is sought which connects the touch to a preceding or following MIDI event. If a connection is found, the touch is assigned the fingering of that MIDI note. If a touch connects uninterrupted to both a preceding and following MIDI note, the MIDI event closer in time is chosen (based on release time of the previous note and onset time of the following note).

**Figure 5:**
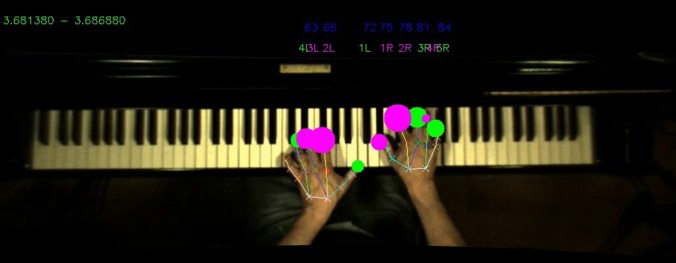
**Screenshot of aligned data sources**. Image is taken from the motion capture camera placed over the keyboard, with MIDI note-on events (blue MIDI numbers), touch event data (green and purple circles), automated fingering of each touch event (green and purple above the keyboard) and motion capture (lines superimposed on each hand) for a segment of time corresponding to the smallest sampling rate (120 fps for the motion capture images).

Following alignment and fingering detection, instructional videos can be rendered showing the camera, marker tracking, touch, and fingering data superimposed (see Figure 5). This method is robust to transient fingering assignment errors, such as a finger passing momentarily above a key while another finger touches the key. The method does not currently handle finger substitutions on a single key press.

#### 2.2.4. Error correction

An advantage of using two or more different types of sensor simultaneously is that each can help mitigate the errors of the other. The two primary limitations of camera tracking are visual occlusion, particularly when the thumb passes under the hand, and limited spatial resolution given the distance needed to maintain the whole keyboard in the frame. Touch data addresses both of these limitations: when the thumb passes underneath the hand, it will typically be in contact with the key, so touch data can be used to maintain knowledge of fingering patterns. Touch sensor data also reports with much finer spatial resolution on each key than the camera.

Conversely, in hot or humid conditions, the capacitive touch sensors can be sensitive to moisture left on the keys from perspiration. Water droplets will sometimes register as small contact area touches even in the absence of a finger. Here the camera data can be used to distinguish genuine from spurious touches. Large contact area touches are nearly always genuine, but when a small contact area touch is noted, its validity can be confirmed by comparing the distance from the distal marker on the camera.

## 3. Method application

This section shows three examples using combined data streams to analyse pianists' movements and actions at the keyboard. These examples are intended to illustrate applications of the measurement method presented in the previous section; a detailed discussion of the musicological implications of the findings is beyond the scope of this paper.

### 3.1. Participants

Four professional pianists situated in Lugano, Switzerland and the surrounding areas were recruited via email. Participants consisted of one female (age 30, pianist 1) and three males (ages 33, 35, and 55, referred to as pianists 2, 3, and 4, respectively), all from Italy. Ethics were followed in participant data collection as set out by the guidelines produced by the British Psychological Society. Participants gave informed consent and were advised that they could abort the experiment at any time, discarding their data.

### 3.2. Materials

Using the setup and alignment processes in Section 2, data was collected from professional pianists' performances of two exercises by Johannes Brahms (no. 13 and no. 40 from *51 Exercises*, WoO 6). The exercises were chosen to demonstrate a pianist's technical finger movement in performing multiple keypresses that require a degree of movement planning and anticipation.

**Figure 6:**
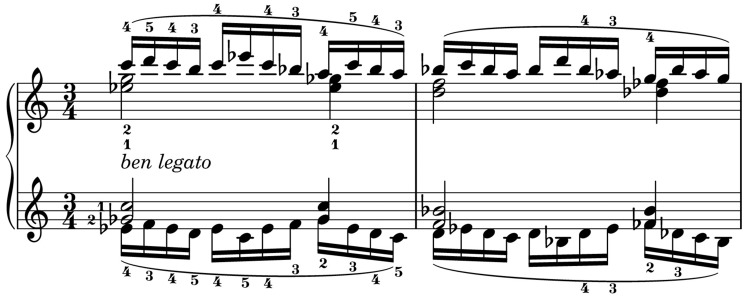
**Brahms Exercise 13 from** ***51 Exercises*****, WoO 6; mm. 1–2**. Fingerings are specified in original.

Exercise 13 (Figure 6) consists of consecutive chords which are held down with the thumb and index fingers of each hand while a sixteenth note melody is played with the middle, ring, and little finger. In the first beat of each bar, the notes in both hands move up and down together in pitch; in beat two they are in contrary motion; in beat three the patterns in each hand are different. The exercise is marked *ben legato*. The sequence is repeated every bar at successively lower pitches. The key signature is C minor and, and the notes in each repetition are always a mixture of white and black notes.

**Figure 7:**
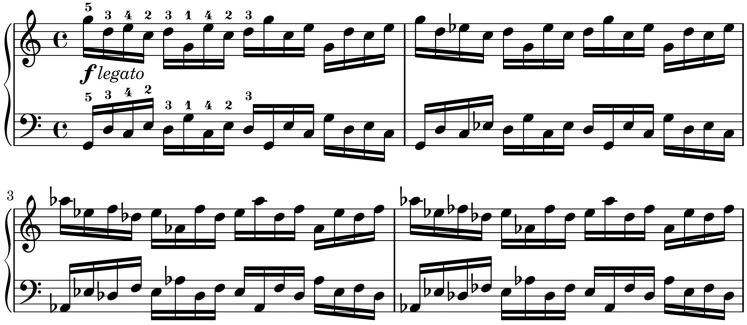
**Brahms Exercise 40 from** ***51 Exercises*****, WoO 6; mm. 1–4**. Fingerings are specified in original.

Exercise 40 (Figure 7) consists of monophonic sixteenth note patterns which require the performer to shift their hands to the right every two bars as the sequence moves up by a semitone. Within each bar, the two hands move in contrary motion. The exercise is played with the marking of *forte legato*. Although the key signature is C major, the chromatic pattern means that the majority of notes in the first two bars use the white keys, the second two bars use predominantly black keys, and this pattern alternates throughout the piece.

An important difference between the exercises is the role of constrained finger motion. In Exercise 13, the third, fourth and fifth fingers are constrained by the need to sustain chords in the thumb and second finger, whereas in Exercise 40, the fingers are free to move, allowing more variation in hand position within each bar.

### 3.3. Data analysis

Analysis is conducted for three distinct cases of finger-key interaction that benefit from the integration of the different sensors, although there are many potential applications depending on the research question. The first case addresses the use of extended (flat) or flexed (curved) fingers to perform a keypress action. In the case of piano performance, this may occur for a number of reasons, either the posture is manipulated in order to produce a certain aural image, or the posture may be changed due to physical constraints concerning the layout of the preceding and proceeding pitches. The second case looks at the touch event in comparison to the onset and release of the MIDI note, revealing both anticipatory and after-touch effects applied by the finger. The third case focuses on the movement transitions between consecutive keypresses using the same finger, where more overall intentions applied to groups of keypresses may be revealed.

#### 3.3.1. Hand posture: extended or flexed fingers

An advantage of the proposed method of data collection and integration is in detecting the position of surface touches in relation to hand movement when the fingers are in a more curved position. As the 2D movement is recorded with an aerial view of the keyboard, we can infer the curvature of each finger joint based on the relative distances between the sets of XY coordinates. For a single touch event, we can assume that the tip of the finger will be in contact with the key (as measured by the touch data) and so this end of the finger can be considered fixed (or at least moving in relation to the key itself). As the finger phalanxes are rigid objects, we can then infer that any decreases in euclidean distance between the coordinates of the various markers of that finger will be due to a flexion or extension of the finger joint.

Based on these assumptions we calculate a *curvature index* (CI) for the distal phalanx and the proximal phalanx. As seen in Equation (1), the CI is a ratio of the distance *d*(*t*) between two sets of XY coordinates at time *t* compared to the same distance *d*_*ref*_ measured at a reference frame when the fingers were laid flat on the keys. For the CI of the distal phalanx, the distance is calculated between distal marker and touch sensor location, using nearest-neighbor interpolation on the touch data to find the point closest in time to the camera frame. For the CI of the proximal phalanx, the distance between the distal and proximal markers is used.

(1) $$CI(t)=\frac{d(t)}{d_{ref}}$$

A CI value of zero in the distal phalanx represents a finger posture where the distal marker is directly above the touch location (fully vertical). A positive CI value reflects a degree of curvature, with a value of 1 representing a fully extended finger (i.e., lying flat on the surface of the key). A negative CI value may occur on the occasion where the distal marker is bent over the touch location.

#### 3.3.2. Surface contacts: anticipatory and release actions

Every key press must be accompanied by a period of finger-key contact. The timing of the touch events in relation to MIDI onset and release times can yield insights into a performer's technique.

The finger is expected to contact the key surface prior to the MIDI note being registered and remain in contact afterward, with the exception of high-velocity notes played with a percussive (struck) touch, where a collision between finger and key may cause the key to separate from the finger on its way down. The removal of the finger from the key surface may occur either before or after the MIDI release event, since the inertia of the key means that the finger can be removed before the key returns to its resting position.

Using the touch sensor data which has been segmented into notes and assigned fingers, we can analyse the relative timing of finger-key contacts vs. MIDI notes as a function of finger, performer and piece. This may be particularly relevant as performers' keypress timings have already been shown to demonstrate large individual differences (van Vugt et al., 2013). In this analysis, for each MIDI note, a contiguous block of touch frames is identified using the segmentation in Section 2.2.3. The touch data is preprocessed to remove spurious touches caused by moisture (Section 2.2.4). In this case, touches with a contact area of less than 20% of the maximum area for that note are discarded, regardless of their location with respect to the marker data. Empirically, this produces a clear distinction between genuine and spurious touches. The first and last notes in the excerpt for each finger are excluded from analysis to eliminate effects related to starting and stopping the performance.

A touch *anticipatory time* is calculated as the first timestamp of this block minus the MIDI onset time; negative values thus indicate the touch precedes the MIDI note. A touch *release time* is calculated as the last timestamp of this block minus the MIDI release time; negative values indicate the finger is removed before MIDI release, positive values that the finger lingers on the key after the MIDI release.

#### 3.3.3. Finger movements: transitions between notes

For transitions between consecutive notes using the same finger, we can analyse the continuous motion of each phalanx of the finger in comparison to the movement of the touch location on each key. The action of releasing or pressing the key can be classified into two categories: *lifts/falls* and *slides*. A *lift* is categorized by a little or no movement at note release, i.e., the finger moves straight up while the touch remains at a fixed point on the key surface. A *fall* is the same motion in reverse during a key press, i.e., the finger moves straight down and again the touch remains at a fixed point. Conversely, a *slide* is categorized by a significant amount of XY movement experienced along the key surface during the release or press of a key.

**Figure 8:**
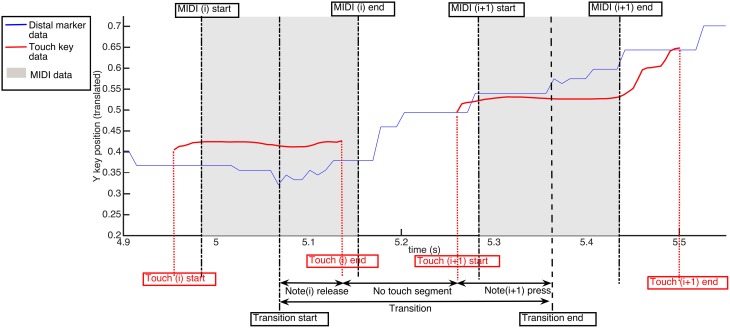
**Timing of transition window between two MIDI keypresses**. Three segments are detailed between any two given keypresses *i* and *i* + 1: note(*i*) release, the no touch segment, and note(*i* + 1) onset. The Quantity of Motion Index (QMI) is detailed for these three segments in comparison to the amount of total distal marker movement over the whole transition window.

In Figure 8, we define a *transition window* between two consecutive notes played by the same finger; the window starts at the midpoint of the first MIDI note (halfway between onset and release) and ends at the midpoint of the second MIDI note. The transition window is divided into three segments: note *i* release, a *no-touch* segment between notes, and note *i* + 1 press. The no-touch segment typically exhibits the largest overall motion as this is the time in which the hand often shifts position to reach the next notes. In fact, the increase in Y position of the distal marker in Figure 8 in the no-touch segment is larger than either the note *i* release or note *i* + 1 press segment. In this example, the touch data in the note *i* release segment and the note *i* + 1 press segment can be characterized as a *lift* and *fall*, respectively. However, the next release segment (note *i* + 1 release) is representative of a *slide*.

For each segment in the transition window, we calculate a *quantity of motion index* for the note release. For example, for the release segment, we have:

(2) $$QMI_{note(i)release}=\frac{QoM_{note(i)release}}{QoM_{transition}}\times \frac{t_{transition}}{t_{note(i)release}}$$

where *QoM_note(i)release_* is the distance traveled for a particular marker during the release phase and *QoM_transition_* is the distance that marker travels during the entire transition. The ratio is time-normalized using the total time of the transition *t_transition_* divided by the time of the release segment *t_note(i) release_*.

### 3.4. Results

We apply the analysis procedures described in Section 3.3 to the collected recordings. The analysis in each section is chosen to demonstrate the breadth of information that can be revealed by the integration of data using the presented method.

#### 3.4.1. Hand posture: extended or flexed fingers

**Figure 9:**
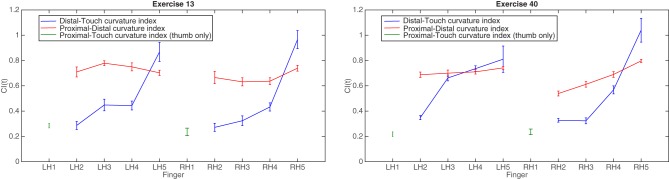
**Group means for distal-touch and proximal-distal curvature index reported for each finger for Brahms Exercises 13 (left) and 40 (right)**. Proximal-touch curvature index reported for each thumb. Error bars are standard error of the mean.

In order to demonstrate differences in the flexion/extension of fingers during the performance of two different pieces, Figure 9 shows the calculated CI categorized by finger for two Brahms exercises. The plot on the left shows mean CI by finger for one professional pianist performing Exercise 13; the plot on the right shows the same CI for the same pianist performing Exercise 40. This allows us to compare CI across two different compositions which may require different hand positions in order to perform the notes. In general there is a tendency for the index finger (LH2 and RH2) to have the lowest distal CI of the four fingers (we exclude the thumb from comparison here as it does not operate in the same manner as the fingers with both proximal and distal phalanxes). This indicates that the index finger has the most flexed distal phalanx and so is more curved when pressing the key. The little finger (LH5 and RH5), tends to have the highest distal CI indicating that in most circumstances, the finger is fully extended to press the key. Comparing across the two pieces, the index, middle and ring fingers of both hands in Exercise 13 have a smaller mean CI for the distal phalanx than in Exercise 40, suggesting that for the Exercise 13, the fingers need to be more curved at the point of key contact than in Exercise 40. This may be expected, as performing simultaneous keypresses such as the first chord of this Exercise 13 will require a hand posture that is curved at all finger joints in order to reach all keys (a mixture of black and white keys). Between hands in Exercise 13, there is a general symmetry of CI across the fingers. To some extent this can also be said for Exercise 40, however, there are cases where the corresponding fingers between hands perform differently. For example, the LH middle and ring fingers (LH3 and LH4) appears to have a higher mean value in the touch-distal relation and a lower mean value in the distal-proximal relation than the corresponding finger in the RH (RH3 and RH4). Looking at each finger across both exercises, there is a tendency for the ring and finger (LH4 and RH4) to be flatter, and for the index finger (LH2 and RH2) to be the most curved.

In piano performance, the choice of using extended or flexed fingers can represent an effect of the physical constraints that arise from the pitch layout of keypresses, but may also indicate the pianist's intention to create certain timbral or dynamic variations in the produced sound. These measurements reveal information about a number of relationships between the fingers on the same hand, across both hands, and between different pieces of repertoire. An advantage of using the comparison between the surface touch location and the distal marker from the camera data is that small changes in location of pressure from the fingertip will be registered even when the position of the finger overall (according to the camera) does not necessarily move.

#### 3.4.2. Surface contacts: anticipatory and release actions

**Figure 10:**
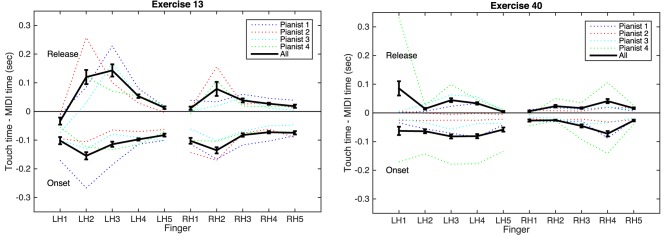
**Differences between touch and MIDI events for Brahms Exercises 13 (left) and 40 (right)**. Top lines in each plot: note releases; positive difference means touch releases after MIDI note. Bottom lines in each plot: note onsets; negative difference means touch begins before MIDI note. Error bars are standard error of the mean.

Figure 10 shows the calculated anticipatory and release timings (i.e., the difference between onsets/releases of the MIDI notes and touch events) for the two Brahms exercises, organized by finger. Variations among players are evident, but some trends are notable. In all cases, the mean anticipatory time is negative, showing that the touch onset precedes the MIDI note. In Exercise 13, for each player, the second (index) finger of each hand exhibits the longest anticipatory time. In this exercise, the thumb and second finger hold long notes; notably, the thumb does not show a long anticipatory time, suggesting that the players locate these keys by first placing the second finger on the key surface and then moving the thumb into place.

The mean release time for most cases is positive, showing the fingers lift from the key surfaces after the MIDI note has concluded. The exception is the left-hand thumb in Exercise 13, which lifts from the surface while the key is still depressed. This suggests that the thumb is rising more quickly than the key can return to its resting position. This could be either an effect of restriking the same note (since each measure contains two notes of the same pitch for the thumb), or it could be a result of achieving the *ben legato* marking by moving the thumb quickly into position on the next key at each new bar.

Exercise 40 shows less clear variation by finger, as might be expected from the score, where every finger has a similar role. Variation across players is more notable here, with one pianist consistently leaving the fingers in contact with the key surfaces for longer times both before and after each note. This demonstrates a difference in technique which could be either a practical or expressive decision on the part of the player.

#### 3.4.3. Finger movements: transitions between notes

**Figure 11:**
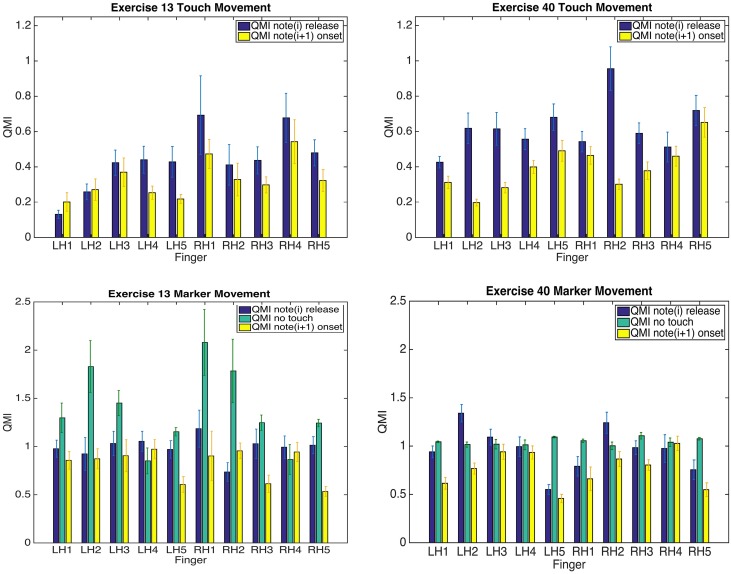
**Means of Quantity of Motion Index for touch movement in note(*****i*****) release and note(*****i*** **+ 1) onset segments in the top-panel of plots, and for distal marker movement in note(*****i*****) release, no-touch and note(*****i*** **+ 1) onset segments in the bottom-panel for Brahms Exercises 13 (left) and 40 (right)**. For each thumb, proximal distance is reported. Error bars are standard error of the mean.

Finally, comparing the amount of movement of the finger with the amount of movement experienced at the key surface, Figure 11 in the top panel shows the quantity of motion index for the touch location for each note release and press event, categorized by finger for one pianist; The same figure shows the corresponding measurements for the distal camera marker for the three segments of each transition in the bottom panel, again for the same pianist. From the touch QMI measurements for both Exercises we can see that in the majority, the keypress action for all fingers is back-loaded, meaning that the majority of the surface movement takes place at the release of the key, in preparation for moving to the next consecutive keypress. Larger QMIs are seen in Exercise 40 than in Exercise 13, suggesting that the legato articulation in the single consecutive notes is achieved by larger slides at touch releases than in the case of held chords. Comparing these results with the distal marker movement, we see a difference again between exercises, where the no-touch segment tends to be larger in Exercise 13 than in Exercise 40. From this we can assume that the majority of movement takes place between the finger key-contact events. This is not so much the case in Exercise 40. In fact, the largest no touch segment movements in Exercise 13 are seen for RH1, RH2, and LH2. As the score (Figure 6) shows, the thumb and index fingers of both hands are playing the held chords throughout the duration of a series of sixteenth notes. The movement in Figure 11 may reflect the larger movements between keypresses that occur due to the whole hand requiring a shift in posture for every chord.

Transition behavior between keypresses can contain information regarding the previous and proceeding events. The anticipatory movements that are used within the touch event show the intention to move toward the next keypress and the difference in Exercises reflects different compositional demands that will have an effect on the transition movement. This in-depth analysis is illustrated for one performer as an example, however, comparisons could theoretically be made between performers ascribing to different piano methods in order to investigate whether this performance style is evident in their movements on the key surfaces and between keypresses.

## 4. Conclusions

This article presents a method of integrating data from complementary sensor technologies: marker tracking from a high-speed camera, touch location measurement with capacitive sensors, and MIDI key press measurements from infrared sensors. Cameras and MIDI sensors are frequently used on their own, but this article shows how connecting subtle actions taking place on the key surfaces with finger motion above the keys can provide novel perspectives on piano performance.

The sensor technologies used in this paper can be distinguished from most existing experimental setups through their focus on the horizontal plane of motion. Measurements of force, key angle, finger height and joint flexion generally examine vertical motion, since this is the axis in which the keys move. However, movements within the plane parallel to the key surfaces are foundational to playing complex passages spanning multiple key presses. In comparison to techniques relying exclusively on cameras, the method presented here achieves greater spatial and temporal detail of finger actions on the key surfaces while reducing problems from occlusion.

This paper presents three example analyses of measurements acquired with the sensor combination method. The finger flexion analysis (Section 3.3.1) relates continuous changes in finger angle to contact location on the key surface. Studies measuring accelerometer-based hand tilt (Grosshauser et al., 2012) or joint angles of each finger (from datagloves Furuya et al., 2011a or motion capture Goebl and Palmer, 2013) are limited to discrete MIDI data in their measurements of key contact (though continuous key angle is used in certain studies Kinoshita et al., 2007). In addition to examining the horizontal plane, touch sensor data offers high spatial and temporal resolution and information about the finger-key contact even when the key is not pressed, which is useful for studying how the performer begins and ends a note (Section 3.3.2).

A related benefit from sensing touch location on unpressed keys is the potential to examine the motion of fingers which are not actively playing a note. Non-striking finger motion analysis has been performed using datagloves (Furuya et al., 2011a; Furuya and Soechting, 2012), but given the important role of tactile feedback in piano performance (Goebl and Palmer, 2008, 2013), touch sensors are valuable for recording the exact time and location of any contacts by the non-striking fingers.

The final analysis (Section 3.3.3) shows that comparing motion of each part of the hand with surface contact location can be useful for studying transitions between successive notes played by the same finger. In particular, small (potentially sub-millimeter) movements at the start or end of one key-press can be compared with the longer action of moving the finger from one key to the next. These comparisons have the potential to yield insight on motor planning in complex passages, where any single sensor modality would not provide sufficient detail.

From a technical perspective, the method also demonstrates how to integrate an array of independent sensors on the device (each TouchKeys sensor) with a single set of continuous sensors on the user's hands (the painted markers). Aligning the temporal and spatial dimensions was the first main challenge of this integration, followed by segmentation and assignment: touch data needs to be assigned to specific fingers, while marker data needs to be segmented into specific notes. Piano performance is the focus of this study, but the method could equally be applied to studies of typing, smartphone usage, or any interface which has a multitude of separate controls. The combination of on-body and on-device sensing allows the researcher to understand the larger movements which enable and connect the manipulation of individual controls.

Extensions of this work could include use of continuous key angle measurements (McPherson, 2013; Bernays and Traube, 2014) in place of MIDI, 3D motion capture systems (Furuya et al., 2011b, 2014; Goebl and Palmer, 2013) in place of 2D camera marker tracking, or integration of force sensors alongside the existing modalities (Parlitz et al., 1998; Kinoshita et al., 2007; Grosshauser and Tröster, 2013).

### Conflict of interest statement

The authors declare that the research was conducted in the absence of any commercial or financial relationships that could be construed as a potential conflict of interest.
